# Multiyear monitoring of threatened iconic arboreal mammals in a mid‐elevation conservation reserve in eastern Australia

**DOI:** 10.1002/ece3.8935

**Published:** 2022-05-24

**Authors:** Ross L. Goldingay, Darren McHugh, Jonathan L. Parkyn

**Affiliations:** ^1^ 4571 Faculty of Science Southern Cross University Lismore New South Wales Australia

**Keywords:** *Petauroides armillatus*, *Petauroides volans*, *Phascolarctos cinereus*, Richmond Range National Park, threatened species refuge

## Abstract

Multiyear investigations of population dynamics are fundamental to threatened species conservation. We used multiseason occupancy based on spotlight surveys to investigate dynamic occupancy of the koala and the greater glider over an 8‐year period that encompassed a severe drought in year 6. We combined our occupancy estimates with literature estimates of density to estimate the population sizes of these species within the focal conservation reserve. Both species showed substantial yearly variation in the probability of detection (koala: 0.13–0.24; greater glider: 0.12–0.36). Detection of the koala did not follow any obvious pattern. Low detection of the greater glider coincided with the drought and two subsequent years. We suggest the low detection reflected a decline in abundance. The probability of occupancy of the koala was estimated to be 0.88 (95% CI: 0.75–1.0) in year 8. Autonomous recording units were also used in year 8, enabling an independent occupancy estimate of 0.80 (0.64–0.90). We found no evidence of a drought‐induced decline in the koala. Habitat variables had a weak influence on koala occupancy probabilities. The probability of occupancy of the greater glider changed little over time, from 0.52 (95% CI: 0.24–0.81) to 0.63 (0.42–0.85) in year 8. Modeling suggested that the probability of colonization was positively influenced by the percentage cover of rainforest. Increased cover of these nonbrowse trees may reflect thermal buffering, site productivity, or soil moisture. We estimate that our study reserve is likely to contain >900 adult koalas and >2400 adult greater gliders. These are among some of the first reserve‐wide estimates for these species. Our study reserve can play an important role in the conservation of both species.

## Graphical abstract

We investigated dynamic occupancy in two iconic arboreal marsupials over an 8‐year period that encompassed a severe drought in year 6. We found no evidence of a drought‐induced decline in the koala population. In contrast, a substantial decline in the detection of the greater glider in the drought year and the two following years suggested a large decline in abundance had occurred.

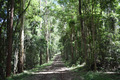

## 1 INTRODUCTION

Population monitoring conducted over multiple years is central to threatened species management enabling population trends, as well as responses to threats and interventions, to be identified (Scheele et al., 2019). For most species there will also be a need to conduct monitoring of multiple populations because environmental conditions and threats may vary geographically. Selection of populations for monitoring needs to consider the phenomenon of site‐selection bias, whereby sites where species are known to be especially abundant are chosen for monitoring (Fournier et al., 2019; Pechmann et al., 1991). Populations are likely to fluctuate in abundance, as well as experience local‐scale colonization and extinction, so those exhibiting high abundance and occupancy when surveys begin may be at a peak in an abundance cycle and subsequent monitoring may be more likely to detect a decline, as those populations decline to a long‐term average. Consequently, concerns will be raised about populations declining in abundance and contracting in distribution. This outcome highlights the need to replicate population monitoring across many locations but specifically to include locations not chosen based on former knowledge of abundance.

Australia is a continent with many unique flora and fauna. However, its endemic land mammal fauna has suffered a disproportionately high rate of extinction relative to other continents (e.g., 28 Australian mammals are extinct compared to one in North America) and there is concern that this will continue based on a further 21% of these species being recognized as threatened (Woinarski et al., 2015). For this reason there is a great need to conduct population monitoring of the Australian mammal fauna, particularly among those recognized as under threat of extinction. The koala (*Phascolarctos cinereus*) is arguably Australia's most iconic mammal (McAlpine et al., 2015). There are serious concerns about its conservation with approximately two‐thirds of its regional populations believed to be in decline (McAlpine et al., 2015). The Australian government recently upgraded the conservation status of the koala from *vulnerable* to *endangered* (DAWE, 2022). The greater glider (now recognized as three taxa with the central species (*Petauroides armillatus*) the subject of this study; McGregor et al., 2020), like the koala (Moore & Foley, 2005), is a highly selective arboreal folivore (Kavanagh & Lambert, 1990; Youngentob et al., 2011). The nominate species (*Petauroides volans*), encompassing the three taxa, is listed by the Australian government as *vulnerable* (TSSC 2016). The threats to the koala are well documented and include habitat loss, disease, dog attacks, vehicle strike and climate change (McAlpine et al., 2015; Santika et al., 2014). The threats driving the decline of the greater glider are not well understood, with declines at some locations appearing enigmatic (Lindenmayer et al., 2011). The greater glider is known to be vulnerable to habitat loss associated with timber production and to fire (Lindenmayer et al., 2011; McLean et al., 2018; Kavanagh, 2000). However, recent studies hypothesize the declines are driven by increases in minimum temperatures (Smith & Smith, 2018; Wagner et al., 2020). A large percentage of the geographic range of both species (koala: 11%; greater glider 28%) were burnt in the 2019/2020 megafires in Australia (Ward et al., 2020), highlighting the need for population monitoring across a broad geographic scale.

The koala is well known to be vulnerable to periods of hot dry weather. Gordon et al. (1988) documented the mortality of up to 63% of a koala population in south‐west Queensland (Qld) following a heat wave during a drought, which included a 12‐day period when the temperature exceeded 40°C each day. Seabrook et al. (2011) estimated an 80% decline in koala abundance in south‐west Qld over a 12‐year period that encompassed the Millennium drought of 2002–2007. Lunney et al. (2012) estimated that heatwaves during a drought killed 25% of the Gunnedah population in north‐west New South Wales (NSW). A recent finding explaining this vulnerability to heat and drought is that koalas are highly dependent on drinking free water (Mella et al., 2020). When koalas were provided with free water they drank during all seasons of the year but more frequently and for a longer duration during hot dry weather (Mella et al., 2019). The response of the greater glider to drought and heat waves is currently unknown, although the number of nights with ambient temperature > 20°C is associated with declines in southern Australia (Wagner et al., 2020). Given the vulnerability to decline by the koala and greater glider there is a need for long‐term monitoring to describe the temporal trend in their populations. This would preferably occur at locations scattered throughout their geographic ranges. Currently, no coordinated program has been established for these species (Ashman et al., in press; DAWE, 2021).

The principal aim of this study was to investigate the population dynamics of the koala and greater glider across a single conservation reserve near the middle of their geographic ranges, over an 8‐year period (2014–2021). We used occupancy modeling to describe the dynamics. A secondary aim was to estimate the size of the populations of the two species in this reserve because population data are fundamental to developing long‐term conservation plans for both species and currently few estimates are available. We combined estimates of occupancy with literature estimates of density to estimate the size of the populations. A severe drought occurred in year six of our study. Based on current knowledge of both species we hypothesize that both our study populations would decline in abundance and contract in distribution as a consequence of the drought. Both species require several years to mature and, at most, produce one young per year (Martin, 1981; Smith, 1969). Therefore, should a drought‐induced decline occur we predict there should be evidence of this for 1–2 years after the drought ended. Given that variation in abundance can produce variation in detection probability (Royle & Nichols, 2003) we also predict that a drought‐induced decline in abundance might manifest as a decline in detection.

## 2 METHODS

### 2.1 Study area

**FIGURE 1:**
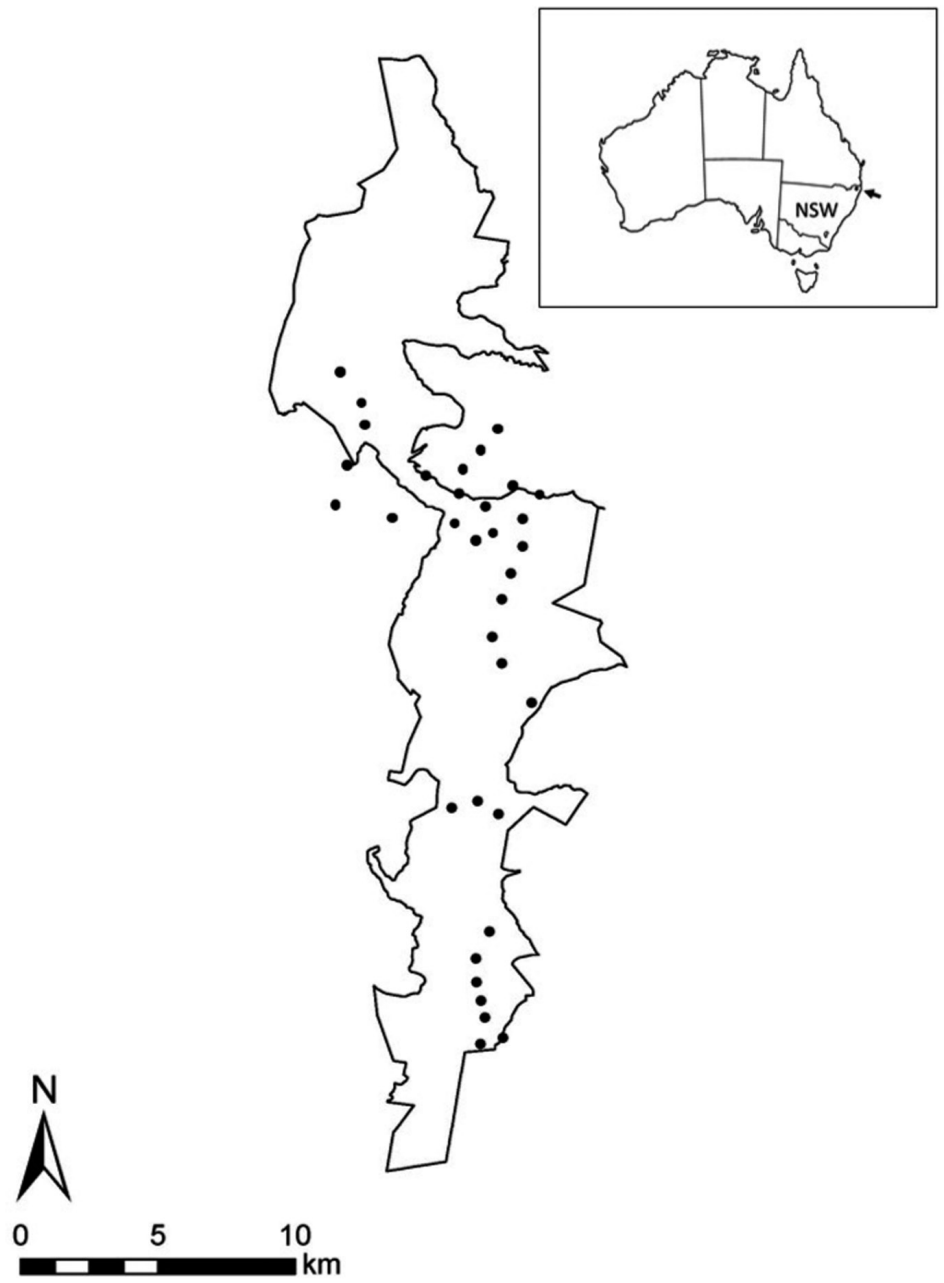
Richmond Range National Park in north‐east NSW, Australia (arrowed on the inset). Solid circles show the location of the survey transects including some in adjoining State Forest

This study was conducted in Richmond Range National Park (NP) (28°43′19″S, 152°44′54″E; 15,657 ha), located ~60 km west of Lismore and ~30 km south of the NSW–Qld border in north‐east NSW (Figure 1). We avoided site‐selection bias because our study area was chosen to monitor another species, the yellow‐bellied glider (see Goldingay et al., 2016), independent of any knowledge of the abundance of the koala and greater glider. The Park contains broad areas of open forest which was dominated by Richmond Range spotted gum (*Corymbia variegata*), flooded gum (*Eucalyptus grandis*), tallowwood (*E. microcorys*), and forest red gum (*E. tereticornis*; authors' personal observations). The open forest is intermingled with large expanses of World Heritage‐listed Gondwana Rainforest. The study area was gazetted as a National Park in 1997. It was previously managed as State Forest and subject to logging.

Annual rainfall at the nearest weather station (Bonalbo Post Office, #57003, 10 km west) averages 1023 mm (Bureau of Meteorology; www.bom.gov.au). Annual rainfall in 2019 (i.e., year 6 of this study) was only 43% of average, and was the lowest in at least 100 years. Rainfall in NSW in the 35‐month period from January 2017 through to November 2019 was the driest 35‐month period commencing in January on record (http://www.bom.gov.au/climate/drought/archive/20191205.archive.shtml#tabs=Rainfall‐deficiencies). Annual rainfall in the 5‐year period before the drought varied between 81% and 103% of average. Annual rainfall was 23%–26% above average in 2020 and 2021.

### 2.2 Survey design

Survey transects of 200 m length were marked out along unsealed roads through the Park where eucalypt forest occurred. We began with 20 transects in 2014 but increased that to 26 in 2016 and to 34 in 2019, by which time they extended across 39 km of forest. Six transects were on roads in adjoining State Forest. Transects were spaced an average of 780 m apart to minimize site dependency. Home ranges of male koalas in northern NSW in forested reserves average approximately 40 ha in size (Radford‐Miller, 2012). Greater gliders have home ranges of 1–5 ha (Kavanagh & Wheeler, 2004; Pope et al., 2004; Smith et al., 2007). Neighboring transects were surveyed concurrently with all transects within a broad area surveyed in succession on the same night so there was no chance of detecting the same individuals on more than one transect in a single survey. Survey sites ranged in elevation from 300 to 610 m. The majority (79%) of transects had experienced no wildfires since 1995. Two experienced a wildfire in 2000/01 and six experienced a wildfire in 2015/16 that did not burn the canopy.

### 2.3 Animal surveys

We conducted surveys three times per year over an 8‐year period, 2014–2021. Spotlight surveys were conducted along each transect over a 20‐min period. Spotlighting was used because it is a general survey technique for nocturnal arboreal mammals (Goldingay & Sharpe, 2004). An element of the survey that related to detection of a species not reported on here was the broadcast of four calls of the yellow‐bellied glider and powerful owl half‐way through a survey to elicit calls from yellow‐bellied gliders. These broadcasts are unlikely to affect detection of koalas or greater gliders. Spotlighting was conducted by a single person who walked at a slow speed (600 m per h) and illuminated forest on both sides of a transect using a LED Lensor P14.2 torch (producing 350 lumens). Any arboreal mammals or nocturnal birds seen or heard calling were recorded. Transects were surveyed on three different nights each year, at least two weeks apart (a maximum of 6 months occurred on one occasion) and typically between September and December, which is the breeding period of the koala when its vocalizations are most frequent (Ellis et al., 2011; Mitchell, 1990). Surveys were conducted usually under ideal conditions of no rain, low moonlight and limited wind, and 1–5 h after dark.

### 2.4 Habitat data

Data were collected within a strip transect that encompassed the 200‐m survey transect and 20 m each side of the road (i.e., 0.8 ha). Greater gliders den in large and typically live hollow‐bearing trees (Kavanagh & Wheeler, 2004; Kehl & Borsboom, 1984; Lindenmayer et al., 1991, 2004; Smith et al., 2007). The abundance of such trees may influence glider occupancy (Eyre, 2006). All live or dead hollow‐bearing trees (HBTs) within the strip were counted as well as the number of trees with a diameter at breast height (dbh) ≥ 60 cm. The height of 10 trees spaced evenly along each transect (one every 20 m) was measured with a laser range finder (Bushnell Pinseeker 1500). We scored the topography (ridge vs. nonridge) of each transect and used a Garmin GPS to record elevation. We retrieved forest type data from the NSW government online portal (NSW government, 2021). We used ArcGIS (ESRI 2011. ArcGIS desktop: Release 11. Redlands CA: Environmental systems Research Institute) to place a 200‐m radius buffer around each transect, to represent the habitat where most animals were detected, and then extracted forest type data within the buffer. We converted data to the % cover of the different forest types within the buffered area. From this we compiled the % cover of preferred food trees of the koala in our area (red gum (*Eucalyptus tereticornis*), tallowwood (*E. microcorys*), flooded gum (*E. grandis*), and Sydney blue gum (*E. saligna*; McAlpine et al., in review); spotted gum (*Corymbia variegata*) and rainforest. We checked for collinearity among all habitat variables. We removed one if the absolute value of the Pearson correlation co‐efficient was ≥.7. The ‘spotted gum’ variable was highly negatively correlated (*r* = −.87) with ‘preferred food trees’. The latter was retained due to its relevance to foraging by koalas. All habitat variables were standardized by subtracting the variable mean from each value and dividing by the standard deviation.

### 2.5 Passive acoustic monitoring

There is no consensus on the best method for detecting koalas with recent studies relying on scat surveys, spotlighting or diurnal searches (Lunney et al., 2017). Some have documented higher detection during spotlighting compared to diurnal searches (Wilmott et al., 2019). Others have documented higher detection from drone‐based thermal imaging compared to spotlighting or scat surveys (Witt et al., 2020). The practical benefits of using autonomous recording units (ARUs) was identified toward the end of our study (Law et al., 2018). In 2021 we installed ARUs to investigate whether our spotlight surveys in that year adequately censused sites occupied by koalas. The koala is a highly vocal species, with vocalizations being most common during the breeding season and given by males >2 years of age (Ellis et al., 2011; Mitchell, 1990). Greater gliders could not be surveyed by ARUs because they do not make loud calls (Henry, 1984). Koalas occupy fixed home ranges (Ellis et al., 2002; Goldingay & Dobner, 2014; Kavanagh et al., 2007; White, 1999) so the detection of calls will indicate whether resident individuals are present in an area (Hagens et al., 2018; Law et al., 2017). We installed one ARU (SM3 or SM4; Wildlife Acoustics) midway along each existing transect during October and December, the breeding season, and concurrent with our spotlight surveys. The ARUs can record over longer periods compared to our spotlight surveys so should provide a much more definitive record of whether sites were occupied. Calls are likely to be detected by the ARUs up to at least 100 m away (Hagens et al., 2018).

The ARUs were programed to record for 5 h after dark. This coincides with the highest period of calling activity of male koalas (Ellis et al., 2011; Hagens et al., 2018; Mitchell, 1990). We installed 12–15 ARUs at a time (i.e., fewer than the total number of transects), using randomly selected site numbers, so there was no spatial bias that might coincide with favorable conditions. We sampled 31 of the 34 transect sites and eight additional sites, that were at least 750 m from an existing site. We resurveyed if malfunctions occurred. ARUs were attached to trees at a height of approximately 1.5 m. They were left in place for 2–3 weeks at each site before being moved to another site. The sampling nights coincided with a period of extreme wet weather, which rendered many nights unsuitable for providing reliable recordings and also made site access difficult. We obtained recordings that were not affected by long periods of rain for five nights at 36 sites and for four nights at three sites.

Recordings were searched for koala vocalizations using the audio software Audacity (Audacity, 2021). Koala vocalizations have a unique spectral signature (Hagens et al., 2018) which enabled identification to be relatively straightforward. Recordings were viewed as spectrograms at a maximum frequency of 4000 Hz with the gain set to 20–35 dB. The times of koala calls were recorded for each 5‐h recording.

### 2.6 Model covariates

We investigated the influence of survey‐specific and year‐varying covariates on detection. Detection of arboreal mammals may be influenced by prevailing weather conditions or moonlight, and may change during the night due to animal activity patterns (Hagens et al., 2018; Law et al., 2018; Wintle et al., 2005). During each survey the time of night was recorded, and the level of wind and moonlight was scored on a three‐point scale. We fitted the following survey‐specific covariates: survey hour after dark, moon brightness (dark, half moon, full moon), wind strength (nil, medium, high), and minimum and maximum temperature on the night of the survey at Bonalbo, the nearest weather station. We also tested the site covariate topography because Law et al. (2018) suggested a weak positive influence of ridge.

We investigated the influence of habitat covariates on other model parameters. These variables were the percent occurrence of preferred koala food trees (red gum, tallowwood, flooded gum, and Sydney blue gum), average tree height, the number of large (≥60 cm dbh) trees, the total abundance of hollow‐bearing trees and topography. The greater glider will also feed in the koala preferred species (Comport et al., 1996; authors' unpublished observations).

### 2.7 Occupancy analyses

We used single‐species multiseason occupancy modeling (MacKenzie et al., 2003) to investigate factors that may influence occupancy dynamics. Multiseason occupancy allows an understanding of dynamic changes in site occupancy (psi, *ψ*) over a series of primary sampling periods (seasons), by including parameters for the probability of colonization (gamma, *ɣ*) and local extinction (eps, *ɛ*), in addition to detection (*p*) which is fundamental to account for imperfect detection (MacKenzie et al., 2003). Modeling was conducted using program presence version 12.24 (USGS Patuxent Wildlife Research Centre, Laurel, MD, 20708, USA). The model estimates the probability of occupancy for the first primary season and the probability of colonization and extinction over subsequent primary seasons (MacKenzie et al., 2003). The probability of occupancy can also be estimated for subsequent primary seasons.

We conducted three surveys in each of 8 years, with years being our primary sampling seasons and the three surveys each year our secondary samples. Multiseason occupancy assumes that the occupancy status of sites (i.e., transects) does not change within a primary season (MacKenzie et al., 2018). Our species are long‐lived and produce no more than one young per year (Martin, 1985; Smith, 1969). They will be resident over multiple years (e.g., Goldingay & Dobner, 2014; Henry, 1984; Mitchell, 1990) so recruitment should occur slowly. We constructed detection histories of all survey occasions (i.e., secondary samples) for all sites to reflect whether each species was detected (1) or not (0), or if a site was not surveyed (–). The latter occurred from time to time due to tree falls and severe erosion that prevented site access, and to account for extra sites added after year two.

We constructed a set of models to test whether covariates influenced our study species. Parameters in a model could be year‐constant, year‐varying, or influenced by a site or sample covariate. Models were compared using Akaike's information criterion for small sample size (AIC_c_), to suggest how well a model explains the data (Burnham & Anderson, 2004). Competing models were ranked from lowest to highest AIC_c_. Differences in AIC_c_ between the model with the lowest AIC_c_ and any other model (∆AIC_c_) suggest the strength of support for competing models (Burnham & Anderson, 2004). Models with ∆AIC_c_ < 2 are considered equally plausible. Increasing values of ∆AIC_c_ indicate less support for a model. If a covariate added to a top model did not improve model fit by >2ΔAIC_c_ it was deemed an uninformative parameter and omitted (see Arnold, 2010). We deleted models that did not converge. We assessed whether there was the lack of fit of the models to the data by using the method of MacKenzie and Bailey (2004). A fully developed multiseason goodness of fit test was not available. Instead, we assessed model fit as implemented in presence with the most general single‐season occupancy model and 10,000 bootstrap samples. The test statistic suggested there was no lack of fit to the data for the koala (*p* = 1.0) or greater glider (*p* = .95).

Model building of the type employed here can lead to some combinations of well‐supported submodels being overlooked if a simple multistage approach is adopted where only the top model from one stage is carried forward to the next stage. To avoid a sequential‐by‐submodel selection strategy (see Morin et al., 2020), we firstly fitted covariates with psi and allowed *p* to vary by year. Models in which ∆AIC_c_ was ≤2 were retained and used to fit models with detection covariates. The top three detection models (≤2∆AIC_c_) were used to fit occupancy models. The top three occupancy models (≤2∆AIC_c_) were used to model gamma and eps. The site covariates were fit to these parameters as well as allowing them to be year‐varying. We specifically investigated whether there was a drought effect by estimating all years before the drought as equivalent and different to the drought year and years after, which were treated as equivalent (i.e., two parameters for gamma and eps).

Detection might be equivalent across years (i.e., null model) or it might differ in some years or all years. We fitted models to determine which scenario prevailed. Because 2019 was a drought year we fitted two detection models to investigate its influence. A drought effect could manifest in the year of the drought or it might arise in that year and continue over subsequent years. In one model, 2019 was estimated as different to all other years, and in another, 2019 and the two years after differed to the rest. To assess an alternative hypothesis that detection differed in some but not all years, we fitted a model with a reduced number of years in which detection differed. We used the output from the fully year‐varying model to determine which years to treat as equal.

We also conducted multimethod occupancy (see Nichols et al., 2008) using program presence to compare the probability of detection of koalas by spotlighting with detection from ARUs in 2021. We used three survey nights for each method, selecting the first three nights of usable audio recordings for each site. Both survey methods involved selecting nights when conditions were most favorable to conduct surveys (i.e., dry and relatively still). The multimethod occupancy model estimates the probability of detection and the probability of occupancy, like other occupancy models, but is structured to estimate the probability of detection from different survey techniques applied at each site. It also estimates an additional parameter referred to as small‐scale occupancy (theta), which is the probability of the species being present conditional on the site being occupied. Audio recording occurred for 5 h so we would expect the probability of detection to be higher with this method but our comparison provides the basis for calibration of the spotlight survey method. We constructed detection histories reflecting whether koalas were detected (1) or not (0), or not surveyed (–) by each method across the three sample occasions (e.g., *H* = 00 01 10). We also conducted single‐season occupancy modeling with only the five‐night audio data to provide simple estimates of occupancy and detection. Models in both analyses were compared using AIC_c_ as described above.

### 2.8 Koala and greater glider population estimates

Our study species are species of immense conservation concern. The koala (excluding the southern part of its geographic range) was recently upgraded to a Federal listing of ‘endangered’ (DAWE, 2022). There are very few population estimates within the endangered range necessitating population estimates and trends within bioregions that could guide conservation efforts to be derived from expert opinion, which was acknowledged to be diverse (Adams‐Hosking et al., 2016). The upgraded status is based on projecting forward from the declines based on that expert opinion. The high level of uncertainty (72%–100%) associated with the NSW population estimates highlights the need for population estimates based on more rigorous methods. There are few population estimates documented for the greater glider (Cripps et al., 2021; Lindenmayer et al., 2011; TSSC, 2016; Vinson et al., 2021) so further estimates will be of value to its conservation.

We used ArcGIS to calculate the area of suitable forest habitat within Richmond Range NP. We combined our mean estimates of the probability of occupancy with literature values of density to estimate population size within the study reserve. We relied on density estimates derived from equivalent forest types and in the same bioregion as our study area. There are numerous density estimates for both species and those used here are at the low end of the range (see Dique et al., 2004; Emerson et al., 2019; White & Kunst, 1990).

We used an estimate of adult koala density based on Law et al. (2022) who used song meter arrays to estimate a density of 0.07 adult males per ha (average of three estimates) in tall forest (Bongil National Park) 170 km south of our study area. The total number of adult koalas can be derived from this based on the adult sex ratio (age class 2 and above). It could be assumed that the sex ratio of adult koalas is 1:1 (M:F; i.e., 0.14 adults per ha). However, several studies have found that populations contain more females than males. Two populations in Victoria had female proportions of 0.9 and 1.8 (Martin, 1985), while three populations in south‐east Qld had female proportions of 1.4, 1.5, and 2.2 (Thompson, 2006). We have used the mean of the five values (1:1.6) to provide an upper adult density estimate of 0.18 adults per ha.

Densities of the greater glider (southern and central species) will vary as a consequence of habitat type and the methods used (Emerson et al., 2019). We used the estimates of Eyre (2006) from south‐east Qld conducted in forest types most similar to those in our study area. We derived a mean value of 0.54 gliders per ha from 10 forest types she sampled. This value includes subadults and adults (males and females). Tyndale‐Biscoe and Smith (1969) recorded detailed data on greater glider population structure in southern NSW. Subadults accounted for 13% of the population. Therefore, we reduced the value of density by this amount to 0.47 per ha to estimate the adult population size.

## 3 RESULTS

### 3.1 Koalas

**FIGURE 2:**
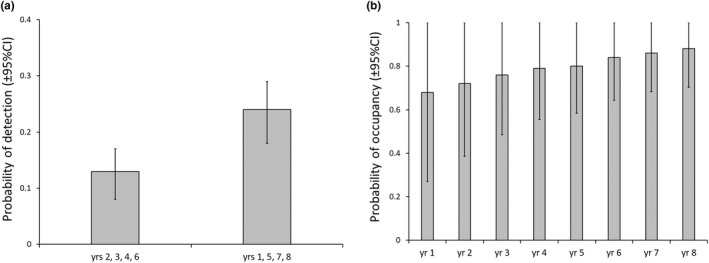
Estimates (mean ± 95% confidence interval) for the koala. (a) Probability of detection showing year (yr) groups, and (b) change in the probability of occupancy over years (yr 1–8). Occupancy is based on model‐average estimates

Koalas were detected 104 times on 82% of transects over the 8‐year study. There was no evidence to support the hypothesis that detection differed in the drought year or in that and the subsequent years (∆AIC_c_ > 8.0). There was no evidence to suggest survey‐specific covariates or that topography influenced detection (∆AIC_c_ > 5.0). There was strong evidence that detection differed among some but not all years (Table 1). Detection was estimated to be lower in years 2, 3, 4, and 6 compared to other years (Figure 2a).

**TABLE 1:** Comparison of the top models for four parameters for the koala in Richmond Range. Three models or those within 2 ∆AIC_c_ are shown for detection (*p*), occupancy (psi), colonization (gamma) and local extinction (eps) parameters. The detection models shown were modeled with psi modeled with ‘Large trees’. Covariates: Year, some years different; min Temp, minimum temperature on survey night; Topography, ridge or other; Large trees, number of trees ≥ 60 cm DBH; Preferred, preferred food trees; HBT, number of hollow‐bearing trees; RF, % cover of rainforest; (.), null model; AIC_c_, Akaike Information Criterion corrected for small sample size; *W*, model weight; ML, model likelihood; *k*, number of parameters

Model (covariates)	AIC_c_	∆AIC_c_	*W*	*k*
*Detection*				
*p*(Year)	552.18	0.00	0.89	6
*p*(min Temp)	557.40	5.22	0.07	6
*p*(Topography)	557.98	5.80	0.06	6
*Occupancy*				
psi(.)	551.45	0.00	0.34	5
psi(HBT)	551.72	0.27	0.30	6
psi(Large trees)	552.18	0.73	0.24	6
psi(Preferred)	553.40	1.95	0.12	6
*Colonization*				
psi(.), gamma(.)	551.45	0.00	0.29	5
psi(HBT), gamma(.)	551.72	0.27	0.26	6
psi(Large trees), gamma(.)	552.18	0.73	0.20	6
psi(.), gamma(Large trees)	552.94	1.49	0.14	6
psi(Preferred), gamma(.)	553.40	1.95	0.11	6
*Local extinction and final models*				
psi(.), gamma(.), eps(.), *p*(Year)	551.45	0.00	0.34	5
psi(HBT), gamma(.), eps(.), *p*(Year)	551.72	0.27	0.30	6
psi(Large trees), gamma(.), eps(.), *p*(Year)	552.18	0.73	0.24	6
psi(Preferred), gamma(.), eps(.), *p*(Year)	553.40	1.95	0.13	6

Occupancy modeled with ‘HBT,’ ‘large trees.’ or ‘preferred food trees’ were equally plausible to the null occupancy model (Table 1). All were used to model colonization. The top colonization model was the null model (Table 1). However, there was evidence to suggest models that included ‘large trees’ modeled with either occupancy or colonization, and ‘Preferred’ modeled with occupancy were equally plausible.

There was no evidence that extinction modeled with any covariate improved model fit beyond the null model (∆AIC_c_ > 2.5). There was evidence that four final models with initial occupancy modeled with different covariates were equally plausible (Table 1). The estimate for eps converged on zero in all models so it was fixed at zero in the final models. To verify whether this was a plausible outcome, we removed the third survey in all years from the detection history and fitted the models from above. There was evidence that the null occupancy model and ‘large trees’ occupancy model were equally plausible but no other models (∆AIC_c_ > 3.0). The model‐averaged estimate of the probability of local extinction based on two surveys per year was 0.01 (95% CI 0.0–0.14). This provides evidence that an estimate of 0.0 for local extinction from three surveys per year is plausible. Model averaging was used to estimate occupancy from the final models (Table 1) across all years of the study. The probability of occupancy varied from a mean of 0.68 in year 1 up to 0.88 in year 8, with evidence based on the 95% CI that there was little change in occupancy over the eight years (Figure 2b). The model averaged estimate of gamma was 0.12 ± 0.08 (±SE).

### 3.2 Acoustic monitoring

Overall, we processed 960 h of recordings over a sum of 192 nights from which we identified 724 bellows of male koalas. On nights when koalas were detected calling rates varied from 0.2 to 5.2 calls per hour. Single‐season occupancy modeling provided evidence (∆AIC_c_ = 5.87) that a model in which detection was equal across the five nights of survey was more plausible than one in which it differed on each night. The probability of detection was estimated to be 0.63 (±95% CI: 0.55–0.71). The probability of occupancy was estimated to be 0.80 (±95% CI: 0.64–0.90).

### 3.3 Spotlighting versus acoustic monitoring

There was very strong evidence (∆AIC_c_ = 25.90) that a model that included detection method (AIC_c_ = 243.04; model weight = 1.0) was more plausible to explain the data than a null model without detection method (AIC_c_ = 268.94). The probability of nightly detection from 5 h of audio‐recording was estimated to be 0.79 ± 0.09 compared to 0.32 ± 0.06 from 20 min of spotlighting. The estimate of small‐scale occupancy was 0.89 ±0.10 and large‐scale occupancy was 0.76 ± 0.07.

### 3.4 Greater gliders

**FIGURE 3:**
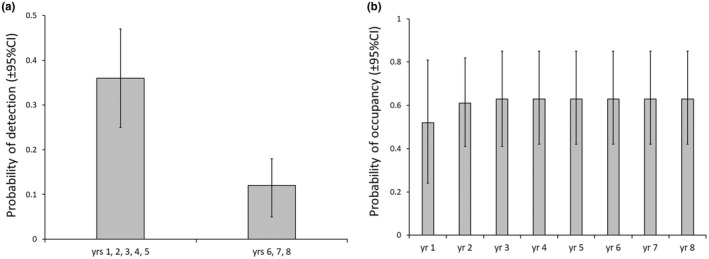
Estimates (mean ± 95% confidence interval) for the greater glider. (a) Probability of detection showing year (yr) groups, and (b) change in population occupancy over years (yr 1–8) in the final model

Greater gliders were detected 118 times on 77% of transects over the 8‐year study. There was strong evidence to support the hypothesis that detection differed in the drought year and the two subsequent years (∆AIC_c_ < 11.0; Table 2). There was no evidence that detection differed in the drought year compared to all others (∆AIC_c_ > 13.0). There was no evidence for the hypothesis that detection was influenced by any survey covariates (∆AIC_c_ > 11.0). The probability of detection was three times higher in the years preceding compared to during or after the drought (Figure 3a).

**TABLE 2:** Comparison of the top models for four parameters for the greater glider in Richmond Range. Three models or those within 2 ∆AICc are shown for detection (*p*), occupancy (psi), colonization (gamma), and local extinction (eps) parameters. The detection models included psi modeled with ‘Large trees’. The occupancy models were modeled with the top detection model. The colonization models are shown with the null occupancy model. Covariates: *D* + 2=drought year and 2 years after; Min*T* = minimum temperature on survey night; Wind = relative wind strength during survey; Large trees = number of trees ≥ 60 cm dbh; Tree height = average height of 10 trees; Elevation = elevation of survey site; RF = percent cover of rainforest habitat; (.), null model; AIC_c_, Akaike information criterion corrected for small sample size; *W*, model weight; *k*, number of parameters

Model (covariates)	AIC_c_	∆AIC_c_	*W*	*k*
*Detection*				
*p*(*D* + 2)	500.23	0.00	0.99	6
*p*(Min*T*)	511.28	11.05	0.00	6
*p*(Wind)	511.79	11.56	0.00	6
*Occupancy*				
psi(.)	499.05	0.00	0.51	5
psi(Large trees)	500.23	1.18	0.28	6
psi(Tree height)	500.78	1.73	0.21	6
*Colonization*				
gamma(RF),	493.21	0.00	0.88	6
gamma(Tree height)	498.27	5.06	0.07	6
gamma(Elevation)	498.90	5.69	0.05	6
*Local extinction and final models*				
psi(.), eps(.), gamma(RF), *p*(D + 2)	493.21	0.00	0.59	6
psi(Tree height), eps(.), gamma(RF), *p*(D + 2)	495.21	2.00	0.37	7
psi(.), eps(Large trees), gamma(RF), *p*(D + 2)	495.37	2.16	0.34	7

Three occupancy models were equally plausible (Table 2). There was little evidence to support other models or a model with both ‘large trees’ and ‘tree height’ (∆AIC_c_ > 2.0). The top three occupancy models were used to fit colonization models. There was strong evidence (∆AIC_c_ < 5.0), regardless of which occupancy model was used, that colonization modeled with ‘rainforest’ was the most plausible model (Table 2). Rainforest had a positive influence on colonization (β = 2.04 ± 1.04). The top extinction model was the null model (Table 2). The extinction models allowed further comparison of the three occupancy models. There was no evidence that ‘large trees’ influenced occupancy (∆AIC_c_ > 4.0) and only moderate evidence that ‘tree height’ influenced occupancy (Table 2). Consequently, a null covariate was used for occupancy and extinction to estimate parameters. The probability of extinction was constant across years and sites 0.29 ± 0.09. The probability of occupancy was estimated from the final model (with rainforest set at its mean) for each year of the study. This value was estimated at 0.52 in year 1. It rose to 0.61 in year 2 and varied little thereafter, although the confidence interval was wide throughout (Figure 3b).

### 3.5 Population estimates

Because forest type did not influence the occupancy of either species we considered all forest types dominated by species of *Eucalyptus* or *Corymbia* as providing suitable habitat. Our study area contained 8684 ha of suitable habitat. The mean estimate of koala occupancy over eight years was 79%. Therefore, we estimate that approximately 6860 ha of forest was occupied by koalas. If the sex ratio of adult koalas is 1:1 (0.14 per ha) then the adult population is estimated to be 960 individuals. However, if the sex ratio is 1:1.6 (0.18 per ha) then the adult population would be 1235 koalas. The mean estimate of greater glider occupancy over eight years was 61%. Therefore, we estimate that approximately 5297 ha of forest was occupied by gliders. Using a density of 0.47 per ha we estimate the adult population to number 2490 greater gliders.

## 4 DISCUSSION

Arboreal mammals are one group of mammals under continuing threat worldwide due to ongoing clearing and fragmentation of their forest habitat. Studies in any geographic region have the potential to inform others in vastly different regions or countries (e.g., Koskimäki et al., 2014). In Australia, long‐term monitoring of koala and greater glider populations is required to better understand factors that drive their population dynamics so any conservation interventions, should they be necessary, can be implemented for these threatened species. Although the koala has been the subject of many studies, few have focused on populations in conservation reserves and involved multiyear monitoring. In NSW most monitoring has combined community surveys with scat surveys (Lunney et al., 1998, 2016). One study combined independent surveys in two periods, mostly 13–22 years apart, to document an 80% decline in occupancy (Lunney et al., 2017). A small number of studies of the greater glider have involved multiyear monitoring. Three of these, spanning 5–22 years, documented substantial declines (Kavanagh, 1988; Lindenmayer et al., 2011, 2021), one spanning 31 years and variable survey effort, also documented a decline (Smith & Smith, 2018), while one spanning seven years documented variable but relatively high abundance (Davey, 1990). These studies of koalas and greater gliders provide many important insights but a clear picture of population dynamics does not emerge. Our study relied on spotlighting, which might be improved with the use of thermal cameras (see Corcoran et al., 2019; Vinson et al., 2020; Witt et al., 2020). Nonetheless, occupancy modeling is designed to deal with imperfect detection and our detection probabilities were adequate. Simulations under a range of sampling regimes show that estimates of occupancy, colonization and extinction are unbiased except at the lower range in the number of replicate surveys and detection values, leading to some overestimation (Mackenzie et al., 2003). In our case this would suggest caution is needed in years when the probability of detection was ≤.10.

### 4.1 Koala dynamics

Our study made several notable findings in relation to the koala. Firstly, occupancy of koalas was very high across our study area and did not vary in response to the drought. Secondly, site variables did not influence occupancy, and thirdly, detection varied across years, although the reasons remain unclear. The probability of initial occupancy was estimated to be 0.68, although it had a wide confidence interval. This estimate is much higher than the naïve occupancy (0.16) recorded by spotlighting at 178 sites located at <800 m elevation by Kavanagh et al. (1995) elsewhere in north‐east NSW. It is similar to that of Law et al. (2018), who estimated occupancy at 0.65–0.75 in north‐east NSW across a range in elevation similar to our study area, and included a small number of survey sites within our study area.

An important finding in our study was that occupancy showed little variation, and appeared to increase as the confidence interval decreased, across the 8‐year study period despite a very severe drought. We predicted that koala occupancy would decline and recovery would be slow, given declines have been observed in koala populations elsewhere during earlier droughts (Gordon et al., 1988; Lunney et al., 2012; Seabrook et al., 2011). We observed no decline and this was not a sampling artifact. We independently verified occupancy in year 8 with ARUs. Occupancy was estimated at 0.80 using the ARUs compared to 0.88 in the same year from the multiseason modeling based on spotlighting.

Another study in north‐east NSW has inferred long‐term stability in a koala population. Lunney et al. (2016) conducted community‐based surveys 21 years apart (1990, 2011) at Coffs Harbour. They also conducted scat surveys at 89 field sites in 1996 and 2011, and found no difference in koala activity. That is, two independent surveys support the hypothesis that the population showed temporal stability over >15 years. Our finding of high koala occupancy over eight years is also surprising given that our study area has a relatively large population of dingoes (McHugh, 2020; McHugh et al., 2019), which are perceived as a serious threat to koalas (see Beyer et al., 2018). We did not investigate this threat but it is noteworthy that koala occupancy increased over a period concurrent with high dingo activity.

Habitat variables had a weak influence on koala occupancy and colonization, and none on extinction. The high level of occupancy suggests high habitat suitability. Three covariate models had equal support to a null model. This may reflect a low sample size relative to variation in these variables. The variables had a slight negative influence on occupancy which may arise because koalas use a variety of tree species other than their primary food tree species (Callaghan et al., 2011; Phillips et al., 2000) and may use, and require, some species and size ranges during the day that differ to those preferred at night (Marsh et al., 2013). The drivers behind the patterns of tree use are complex (Moore & Foley, 2005; Moore et al., 2004) and may not be readily captured by the variables measured here.

Detection was low in four of the eight survey years. Rainfall varied among years but not consistently with detection. We predicted that should the drought induce a decline in abundance it might manifest as a lower detection probability. We found no evidence of this. The reasons for the heterogeneity in detection are unclear. Law et al. (2018) observed yearly variation in detection but implicated a change in the model of ARU. They found that detection based on calling declined as minimum temperature increased. They related this to a seasonal change in calling with lower calling at the end of the breeding season in December when temperatures are higher. We found no influence of minimum temperature. This may reflect that 25% of our site detections were visual detections from eyeshine.

### 4.2 Great glider dynamics

Our study made several notable findings in relation to the greater glider. Firstly, we recorded a substantial decline in detection (from 0.36 to 0.12) coinciding with a record drought. Secondly, site variables had a relatively weak influence on initial occupancy. Thirdly, colonization was influenced positively by the amount of rainforest on a transect. The probability of initial occupancy was estimated to be 0.52. This compares to an estimate of 0.51 by Wintle et al. (2005) in south‐east NSW. There are no other estimates of occupancy available that have allowed for the probability of detection. Kavanagh et al. (1995) recorded greater gliders at 51% of 291 survey sites in north‐east NSW. Greater gliders are expected to be “easy to detect” (Smith & Smith, 2018). Wintle et al. (2005) estimated a single‐visit mean value of 0.41 within a single year. This is close to our estimate of 0.36 before the drought. Cripps et al. (2021) used a double‐observer method to estimate the probability of detection by one observer conditional on the animal being detected by another, which was 0.64. This measure may not be directly comparable to repeat surveys conducted several weeks apart but provides a useful measure of repeat detection on the same night. Our modeling estimated detection to be substantially lower (0.12) in the drought year and the two subsequent years. Heterogeneity in detection is likely to be closely related to variation in animal abundance (Royle & Nichols, 2003). Therefore, we suggest that the decline in detection in the last three years of our study reflects a decline in abundance which was not reflected in our estimates of occupancy. We speculate that the decline in detection was a consequence of the drought but we cannot rule out other causes. The greater glider has a low reproductive rate (Smith, 1969) so if a substantial decline occurred in one year we would predict recovery would take several years. A drought‐induced decline would be consistent with the finding that greater gliders are vulnerable to an increasing measure of aridity (Wagner et al., 2020).

The finding that site variables had a weak influence on initial occupancy is surprising. Wintle et al. (2005) reported a strong influence on occupancy of site quality derived from a predictive model. Habitat quality might be more likely to induce variation in abundance rather than occupancy of greater gliders. Indeed, an increase in the number of hollow‐bearing trees has been found to influence the abundance of greater gliders (Eyre, 2006; Lindenmayer et al., 1990; Vinson et al., 2021). Elevation has been found to influence greater glider occupancy and persistence, with high elevation offering more suitable habitat (Smith & Smith, 2018; Wagner et al., 2020). An increase in temperature over time, particularly the number of hot nights, and aridity (i.e., drought‐like conditions), have been implicated as the causes of this pattern. We found no influence of elevation, albeit over a narrower range (300–600 m) than in earlier studies (80–1000+ m).

The amount of rainforest on a transect had a positive influence on greater glider colonization. This was unexpected given all species of greater glider feed almost exclusively on eucalypt foliage (Comport et al., 1996; Cunningham et al., 2004; Kavanagh & Lambert, 1990). Therefore, rainforest trees do not provide food for this species. Indeed, none of our 118 observations of greater gliders were in rainforest trees. Lindenmayer et al. (2011) reported an association of greater gliders with rainforest, as well as eucalypt forest. The influence of rainforest may arise from such sites having higher site productivity or moisture levels, or may be due to rainforest trees typically having a denser canopy and, therefore, providing thermal benefits to gliders sheltering in hollows in neighboring eucalypts. These suggestions require further investigation because they may give insights to future management of greater glider habitat.

### 4.3 Population estimates

Understanding population size is important for conservation because size can suggest resilience or vulnerability. We estimated the size of the koala population in Richmond Range NP to be >900 adult individuals. The size of few koala populations in NSW has been estimated. Estimates range from 75–347 individuals (all ages) within habitat remnants of 188–856 ha (Crowther et al., 2021) to 350–800 koalas (including subadults) across 4000 ha of forest (Lunney et al., 2004; Matthews et al., 2007). In south‐east Qld, a population of >6000 individuals (all ages) was estimated across an area of 37,400 ha (Dique et al., 2004). The koala population in Richmond Range is connected to State Forest and another protected area that also provides suitable habitat, so the broader population will be even larger. Many if not most koala populations occur as metapopulations (e.g., Close et al., 2017; Norman et al., 2019).

We estimated the size of the greater glider population to be at least >2400 adult individuals. There are few population estimates for any of the species of greater glider. An endangered population of southern greater gliders (*P. volans*) in NSW was estimated to consist of 335 individuals (all ages; Vinson et al., 2021). Cripps et al. (2021) estimated a population of 24,575 (all ages) individuals of the southern greater glider across 25,865 ha in the Strathbogie Ranges of Victoria. Emerson et al. (2019) estimated a mean density of 1.36 per ha within an area of 260 ha, suggesting a population of at least 354 individuals (all ages) of the central greater glider.

Our population estimates suggest large populations of both species occur in Richmond Range NP. The density estimates used are at the lower end of estimates for these species and are based on estimates from the same bioregion as our study area. Mean density is commonly reported to be >0.2 per ha in koalas (Dique et al., 2004; White & Kunst, 1990) and >0.8 per ha in greater gliders (Emerson et al., 2019). Further research in Richmond Range NP should be directed at estimating density to confirm or revise the values here, using distance sampling for the greater glider (Cripps et al., 2021; Emerson et al., 2019) and arrays of audio recorders for the koala (see Law et al., 2022).

## 5 CONCLUSIONS

Understanding multiyear dynamics is central to conserving populations of threatened species. In our study area, occupancy of the koala was high and increased over time (0.68–0.88), whereas occupancy of the greater glider varied little over time (0.52–0.63). We found no evidence that a very severe drought in year six had a negative influence on the koala population. This suggests our study reserve may be an important climate refuge (see Reside et al., 2019) for this species. In contrast, there was evidence that the drought negatively affected the greater glider population. Its detection probability during and after the drought was reduced to one third of predrought estimates. We suggest this was reflective of a decline in abundance or a dramatic change in habitat use. This observation is consistent with those of other authors that suggest the greater glider is sensitive to drought‐like conditions. This may suggest that our study reserve has insufficient elevation to be a climate refuge for this species. Nonetheless, our estimates of the size of the populations of both species suggest our study reserve can play an important role in the conservation of both species and further study of these populations is warranted.

## AUTHOR CONTRIBUTION

**Ross Goldingay:** Conceptualization (lead); Data curation (lead); Formal analysis (lead); Investigation (lead); Methodology (lead); Project administration (lead); Writing—original draft (lead); Writing—review & editing (lead). **Darren McHugh:** Investigation (supporting); Writing—original draft (supporting); Writing—review & editing (supporting). **Jonathan Parkyn:** Investigation (supporting); Writing—original draft (supporting); Writing—review & editing (supporting).

## CONFLICT OF INTEREST

The authors have no conflicts of interest to declare.

## Data Availability

Data used for analysis in this study are accessible at Dryad (https://datadryad.org/stash/share/yCkaPY3nJ3MG2D3i82A_xMvqCjgSm7jYkVpTn‐IzeQ4).
